# Localising functionalised gold-nanoparticles in murine spinal cords by X-ray fluorescence imaging and background-reduction through spatial filtering for human-sized objects

**DOI:** 10.1038/s41598-018-34925-3

**Published:** 2018-11-08

**Authors:** Florian Grüner, Florian Blumendorf, Oliver Schmutzler, Theresa Staufer, Michelle Bradbury, Ulrich Wiesner, Tanja Rosentreter, Gabriele Loers, David Lutz, Bernadette Richter, Markus Fischer, Florian Schulz, Swantje Steiner, Martin Warmer, Anja Burkhardt, Alke Meents, Matthew Kupinski, Christoph Hoeschen

**Affiliations:** 1 Universität Hamburg and Center for Free-Electron Laser Science, Luruper Chaussee 149, 22761 Hamburg, Germany; 2 Department of Radiology and Molecular Pharmacology Program, Sloan Kettering Institute for Cancer Research, New York, New York 10065 United States; 3 Department of Materials Science and Engineering, Cornell University, Ithaca, New York 14850 United States; 4 Helmholtz Zentrum München GmbH, German Research Center for Environmental Health, Institute of Radiation Protection, Ingolstädter Landstraße 1, 85764 Neuherberg, Germany; 5 Center for Molecular Neurobiology Hamburg, University Medical Center Hamburg-Eppendorf, Martinistrasse 52, 20246 Hamburg, Germany; 6 Hamburg School of Food Science, Institute of Food Chemistry, University of Hamburg, Grindelallee 117, 20146 Hamburg, Germany; 7 Institut für Physikalische Chemie, Universität Hamburg, Martin-Luther-King-Platz 6, 20146 Hamburg, Germany; 8 University Medical Center Hamburg-Eppendorf, Martinistrasse 52, 20246 Hamburg, Germany; 9 Photon Science, DESY, Notkestraße 85, 22607 Hamburg, Germany; 10 Center for Free-Electron Laser Science, DESY, Notkestrasse 85, 22607 Hamburg, Germany; 11 College of Optical Sciences, The University of Arizona 1630 E. University Blvd, Tucson, AZ 85719 United States; 12 Institute for Medical Technology, Otto-von-Guericke-University Magdeburg, Universitätsplatz 2, 39106 Magdeburg, Germany

## Abstract

Accurate *in vivo* localisation of minimal amounts of functionalised gold-nanoparticles, enabling *e.g*. early-tumour diagnostics and pharmacokinetic tracking studies, requires a precision imaging system offering very high sensitivity, temporal and spatial resolution, large depth penetration, and arbitrarily long serial measurements. X-ray fluorescence imaging could offer such capabilities; however, its utilisation for *human*-sized scales is hampered by a high intrinsic background level. Here we measure and model this anisotropic background and present a spatial filtering scheme for background reduction enabling the localisation of nanoparticle-amounts as reported from *small*-animal tumour models. As a basic application study towards precision pharmacokinetics, we demonstrate specific localisation to sites of disease by adapting gold-nanoparticles with small targeting ligands in murine spinal cord injury models, at record sensitivity levels using sub-mm resolution. Both studies contribute to the future use of molecularly-targeted gold-nanoparticles as next-generation clinical diagnostic and pharmacokinetic tools.

## Introduction

Therapeutic approaches to pathological disorders would benefit strongly from high-resolution/high-sensitivity *in vivo* imaging and/or pharmacokinetic information. There has been a tremendous surge in both molecularly-targeted probe and device technologies for nanomedicine, which promise to enable earlier, more sensitive and precise detection for various pathologies^1–4^. For example, as a result of the aging of the human population, cancer is the most common cause of death worldwide, and secondary prevention methods, directed at early cancer/metastases detection, will improve the likelihood of successful therapy and higher survival rates^5^. Another opportunity to foster the development of targeted therapy is *in vivo* tracking of medical drugs through the body, based *e.g*. on antibody reactions, *i.e*. pharmacokinetic (PK) studies^6–8^.

One such promising non-invasive imaging solution is based on X-ray fluorescence imaging (XFI) of gold-nanoparticles (GNPs), which are either functionalised (for diagnostics and image-guided radiotherapy) or bound to medical drugs (for PK). The method presented here is expected to offer effectively higher detection sensitivity than Magnetic Resonance Imaging (MRI) as XFI signals only arise from GNP-localisation, are robust against physiological inhomogeneity, and do not require a pre-contrast-injection measurement. The spatial resolution of XFI achievable for human use is in the range of 1 mm or below, defined solely by the applied X-ray pencil beam diameter. This resolution is roughly equivalent to that offered by clinical MRI, but higher than that associated with clinical Positron Emission Tomography (PET) scanners; the latter modality has a typical clinical resolution of 4–5 mm. A key advantage of XFI over PET can be seen in the possibility to frequently monitor response assessments over *arbitrarily long* time periods at low cumulated doses, whereas these conditions would not be practically achievable with PET imaging, given the typically short physical half-lives of radiotracers and the associated higher costs of these studies. In addition, PET is hardly suitable for imaging on short time-scales (below minutes) for large investigation areas due to noise limitations.

The *in vivo* localisation of GNPs, however, depends on the unambiguous detection of emitted *X-ray fluorescence* photons following excitation with incident X-ray photons. The challenge is to provide sufficiently high sensitivity to detect and quantitate very low GNP-amounts within target tumour tissues and other critical organs, particularly at sufficient *in vivo* penetration depths. To achieve this, two independent challenges, both addressed by this work, have to be met, enabling successful deployment of XFI as a clinical tool: (i) the effective surface-functionalisation of GNPs to target disease and (ii) a dedicated scheme needed to distinguish emitted fluorescence X-ray photons from the vast number of *background* photons, especially in case of large objects. Although the XFI-approach has been under investigation for quite some time^9–15^, most approaches still suffer from this intrinsic “*background problem*” for *human*-sized objects at tolerable dose-levels, when the GNP-amounts are as low as expected for clinical applications and/or reported from small-animal studies.

We address the sensitivity issue for applications on *human*-sized scales and show that the use of X-rays with high brilliance, in combination with advanced spatial and spectral filtering, leads to the required reduction of XFI-background. This intrinsic background is predominantly caused by multiple Compton scattering events that lower the energy of the incident photons down to the spectral range of the gold-fluorescence lines. The larger the object, the larger is this intrinsic background effect.

In contrast to brilliant X-ray radiation from *large*-scale synchrotron facilities, the emergence of compact, laser-based X-ray sources^16–19^ supports the possibility for transferring our XFI-method into *clinical* applications with sufficiently short scanning times.

This work is structured as follows: first, we report on a concrete basic research study on functionalising GNPs for the use in murine spinal cord injury models, demonstrating high levels of sensitivity and specificity as well as sub-mm spatial resolution. The second part addresses the issue of the *background problem* in case of larger objects, here a 30cm-diameter tissue-equivalent phantom. We first report on numerical simulations of the Compton-background in full 4π and then discuss our spatial filtering scheme supported by different experimental validation studies as well as an analytical model for explaining the background distributions. We have chosen experiments with mono-chromatic and polarised synchrotron X-ray beams as well as with unpolarised beams from a polychromatic X-ray tube, in order to study the comparison of our model with both types of sources. We also discuss the bandwidth limit of our scheme as this determines the applicability of possible X-ray sources.

## Results

### Localisation of L1-functionalised gold-nanoclusters in murine spinal cords

**Figure 1:**
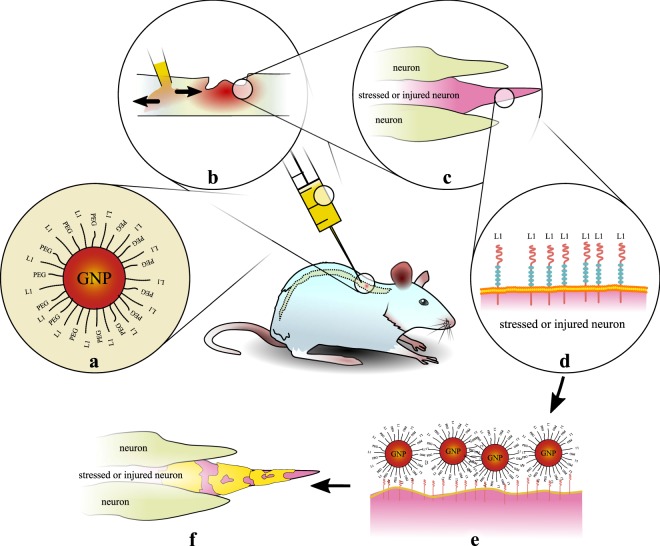
Overview of experimental pilot study towards precision pharmacokinetics. Gold-nanoparticles functionalised with L1-peptides (**a**) are injected into an injured murine spinal cord (**b)**, where stressed/injured neurons are present (**c**). These neurons express L1-proteins on their surface (**d**), to which the L1-functionalised GNPs bind (**e**). By an XFI-scan the regions with bound GNPs have been localised (**f**, see also Fig. 2c).

As a pilot study towards XFI-based PK studies, we first present results of experiments using gold L-shell excitation XFI in murine models with spinal cord injury, and their subsequent treatment with GNPs functionalised with a short peptide derived from the neuronal cell adhesion molecule L1 and thiolated poly(ethylene glycol) (PEG)^20,21^ see Fig. 1.

We assessed the diffusive behaviour of L1-GNPs using a clinically relevant *in vivo* model of spinal cord compression^22–25^. To this end, thoracic spinal cord injury was performed on adult female mice using well-established paradigms. L1-GNPs were applied 500 µm proximal to the lesion site via an ultra-thin capillary to stimulate neural regeneration. PEGylated GNP probes were used as controls. Following serial assessments as long as over a 48-hour time interval (*i.e*., 0, 2, 48 hours), injured mice were transcardially perfused, and the spinal cords subjected to XFI using highly-brilliant synchrotron radiation. Mock-injured spinal cords in the absence and presence of L1-GNPs were used for comparison. The aim here is to explore the sensitivity limit of XFI with respect to detecting an unprecedented minimum amount of locally confined tissue-bound biomarkers of high specificity, in addition to achieving sub-mm spatial resolution.

**Figure 2:**
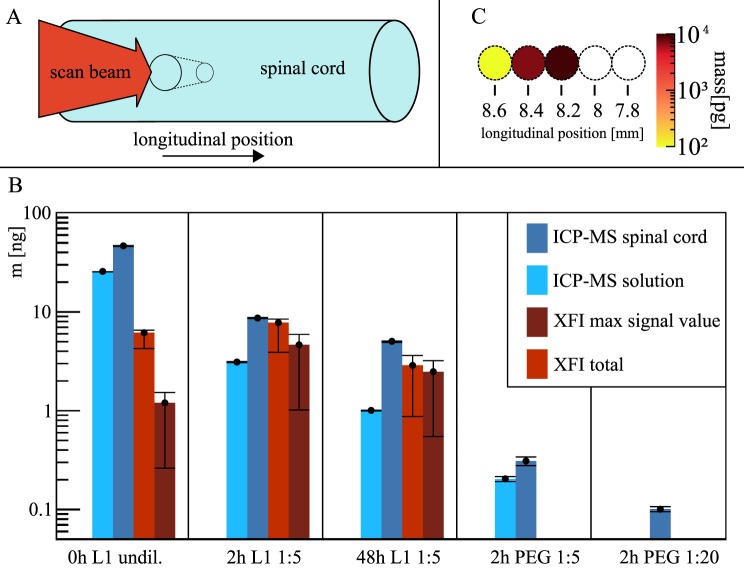
Detection of GNP-amounts in murine spinal cords with XFI vs. ICP-MS. (**A**) Measurement geometry for XFI-scans with scan beam perpendicular to the spinal cord. (**B**) GNP mass, *m* (in nanogram), for the various probes differing in the functionalisation of the GNPs, *i.e*. with L1 peptide and PEG (labelled as “L1”) or only PEG (“PEG”), the post-injection time (from 0, 2, and 48 hours) and the degree of dilution (undiluted, 1:5, 1:20). No XFI-signals were found for the PEG-only cases. For ICP-MS, also the solutions containing the spinal cords were examined. For XFI, the maximum signal value over all scanned positions is given. (**C**) A typical (projected) spatial distribution of GNPs along a spinal cord (white circles indicating no gold-signal) shows a sharp localisation of the GNPs within a fraction of 1 mm. Note that ICP-MS cannot provide such spatial information. The ICP-MS error-bars are from the statistic over 3 consecutive measurements, the XFI error-bars include the uncertainty of the GNP-position along the scanning beam direction.

We detected local GNP-masses down to 72 picogram in the scanning pencil beam volume with a diameter of only 0.2 mm (Fig. 2A).

The statistical significance *Z* (in units of one standard deviation) of this record minimum detection limit in GNP-weight (*i.e*., 0.0017 wt% or weight-percentage) was *Z* = 4.3. The main aim of this experiment was to explore the XFI sensitivity limit in the localisation of GNPs, but we also benchmarked the XFI-detectable local GNP masses against *inductively coupled plasma mass spectrometry* (ICP-MS). Uncertainties in the XFI measurements have been thoroughly studied, and the results from ICP-MS and XFI are in close agreement, noting that no spinal cord was completely scanned, leaving the XFI-signal below ICP-MS values (Fig. 2B). The findings imply that for all post-injection times the entire GNP-mass remains concentrated in the region around the lesion positions, indicating a stable binding of the L1-functionalised GNPs to stressed neurons. On the basis of the XFI-scan obtained at those positions, we deduce an uptake of 0.6% to 1.0% of the total injected GNP-amounts. The transverse scanning (in one plane) along the spinal cords (Fig. 2A) demonstrated strong localisation of the L1-functionalised GNPs at the injection positions (Fig. 2C), an information not available from ICP-MS measurements.

Since the isotropic XFI-signal was measured by a detector with an extremely small fraction of the entire (4π) solid angle, the applied dose was found to be very high. Setting the physical dose limit to 11 mGy (corresponding to 10^6^ incident photons) for 4π-detection and a statistical significance limit of *Z* = 3, we find a minimum detectable GNP-mass of 2.3( ± 0.7) ng, corresponding to 0.055 wt%. These computed GNP-mass and concentration values are effectively more than one to two orders of magnitude lower than those previously published, taking into account the much smaller scanning beam diameter and the use of significantly lower dose levels than used for other probes, especially in XFI-Computed-Tomography (XFI-CT) schemes (ref.^26^ and references therein). The critical factor needed to attain high XFI-sensitivity for the low dose administered was the usage of highly *brilliant* X-ray beams, whose mono-chromaticity allows for quasi background-free measurements.

### Solution of “background problem” for large objects

**Figure 3:**
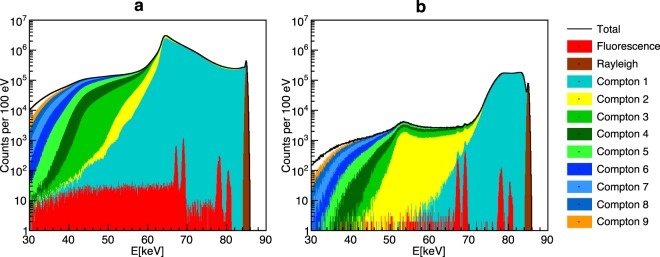
Simulated X-ray spectra demonstrating effective background reduction. A colour-coded logarithmic spectrum for a case with Z = 13 and 1.3·10^9^ incident photons is shown for *full* solid angle detection (**a**) and the optimised spatial filtering (**b**). Fluorescence counts are shown in red, whereas the colour of “Compton *i*” represents the photons undergoing Compton scattering *i*-times. The two fluorescence lines (around 69 keV) are not detectable in the left panel due to the high background level, which our filtering scheme reduces by a factor of 570, making the XFI-lines visible (right panel).

While the above L-shell XFI-experiment demonstrated a quasi background-free setup, its relatively low photon energy makes it impractical for *in vivo* imaging of objects larger than small animals. On the other hand, K-shell XFI in *human*-sized objects is expected to suffer from a large intrinsic background^27–29^ (Fig. 3a).

We have set up analytical and numerical studies of the signal and background behaviour. In order to minimise intrinsic background contributions to the measured signal without concomitant signal losses, a scheme utilizing advanced spatial and spectral filtering was developed, derived from the *anisotropy* of the background. In this work we treat the anisotropy in case of *human*-sized objects, both for monochromatic polarised and polychromatic unpolarised X-ray photons, both in numerical and experimental studies, and provide an explanation from an analytical model.

In order to address size scales relevant for future XFI-applications on *human* patients, we simulated a (soft-tissue) sphere with a diameter as large as 30 cm. In its centre, a small sphere with radius of only 0.5 mm has been placed containing gold at the same concentration of 0.23 wt% as that reported in XFI-experiments using tumour-bearing *mice*^26^, *i.e*. from a *small*-animal model. However, this reported concentration was detected (with a polychromatic benchtop X-ray source) in a cubic voxel of length 2.5 mm, while here we aim to detect the same *concentration* in just a 1-mm-diameter *sphere* - a volume, and hence, GNP-*mass*, a factor of 30 times smaller, *i.e*. in our case only 1.2 μg. Note that the scheme in ref.^26^ could *not* detect this GNP-amount in a 30-cm-object. In our simulation, the large-size phantom was scanned with a pencil beam of 1 mm diameter, defining the spatial resolution of 1 mm, with an average *physical* dose, *i.e*. absorbed energy within the scanning beam volume, set to be only 10 mGy.

**Figure 4:**
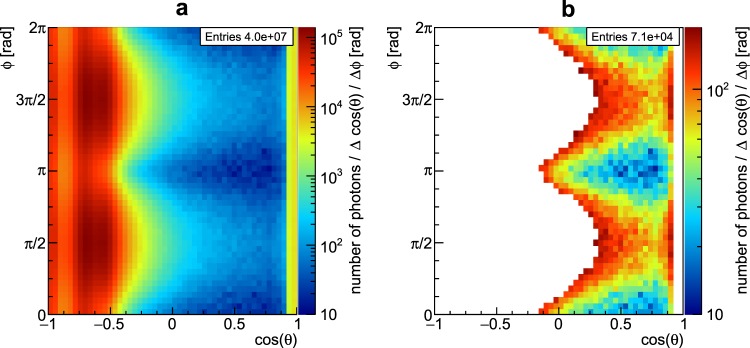
Spatial distribution of the strongly anisotropic Compton background and result of spatial filtering. In (**a**) no spatial filtering is applied, while (**b**) shows spatial filtering exhibiting the highest overall signal significance with the optimum of 85 keV incident energy, using spherical detector coordinates. One can also see the influence of linear polarisation in the azimuthal angle Φ (Φ and cos(Θ) are given with respect to the beam direction). Note that the colour scale varies from (**a**) to (**b**) by almost three orders of magnitude, with the total entries reduced by a factor of 570. A white colour means “discarded pixel”, i.e. such a pixel is removed from contributing to the sum spectrum.

Our ability to determine both the photon detection scheme with maximum background reduction and optimum incident photon energy is based upon an analysis of the energy-dependent spatial distribution of the multiple Compton scattering background. Since we propose to quantify the signal quality as the combined significance of both the Kα_1_ and Kα_2_ lines of the GNPs, we apply *spectral* filtering around these two lines (within a range of ± 3σ_det_ around each line, with a detector energy resolution of σ_det_). The manifold of all possible combinations of multiple Compton scatter angles with final energies falling into the range around these two fluorescence lines results in a strongly *anisotropic* background (Fig. 4a).

Due to this *anisotropic* background in *large* objects, a detector covering the full solid angle - similar to the 4π-detection extrapolation of our spinal cord measurements - does not automatically imply an optimised setup (see the corresponding spectrum in Fig. 3a), despite its maximum *signal* counts.

The two basic constraints, which our detection scheme fulfils, are to (i) allow the GNP-loaded subvolume to be *anywhere* along the scanning beam volume *and* (ii) exclude higher-order Compton scattered photons from the scanning pencil beam volume. The first constraint, aimed at maintaining a minimum dose level and time by requiring only a *single* exposure per scanning direction, rules out any collimators for which GNP position must be known *a priori*, as assumed *e.g*. in ref.^30^ or our own previous work^31^. The second constraint is realised by using a set of molybdenum leaves, radially arranged around the beam, such that detected photons can only arise from within the scanning beam volume, effectively excluding higher-order Compton photons. The simulated X-ray beam is linearly polarised and chosen to be monochromatic, as would be expected from synchrotron or laser-driven sources – two principle sources that allow a sufficiently fast XFI-scanning time.

The solution to the “background problem” is to consider it as an optimisation problem. To that end, the entire cylindrical detector surface is divided into a set of pixels. For any given incident photon energy, our algorithm determines that subset of pixels whose sum spectrum exhibits the maximum statistical signal significance *Z*. The single spectrum of each pixel alone does not show a significant signal, hence the spectra of many pixels are added to a sum spectrum. Our algorithm discards those pixels with the largest yields from the adding to a sum spectrum until this sum spectrum shows a statistically significant signal. Once this signal is found, the algorithm then removes further pixels as long as the resulting significance of the remaining sum spectrum reaches a maximum. We have found that this algorithm proves to be robust for arbitrary locations of the gold-distributions inside the object. The sum spectrum of this optimal subset allows the detection of the desired small gold concentration of 0.23 wt%, *i.e*. 1.2 μg, with a minimum statistical significance limit of *Z* = 3. We have tested our algorithm with many different incident photon energies and have found that 85 keV delivers the maximum significance Z over all other incident energies. This finding of optimum incident photon energy close to the K-edge energy of gold can be understood in terms of our optimisation solution: the smaller the difference between incident photon energy and the K-edge, the more dominant is *single* Compton scattering, while higher order Compton scattering becomes more important at higher incident energies. The background of *single* Compton scattering exhibits the highest degree of anisotropy, which is the basis for the solution presented here. Thus, at higher incident energies, higher Compton order scattering leads to a less and less anisotropic background, precluding the presented spatial filtering.

Figure 4b shows the result of our solution for a polarised X-ray source. If one would use an unpolarised source, the “wave-like” pattern would be transformed into Φ-independent vertical columns. However, we have simulated both cases and have seen that Z_pol_ = 13.62( ± 0.22) and Z_unpol_ = 13.66 ± (0.33).

In order to decrease radiation dose and exposure time, our scheme does not require a full CT because particle localisation can simply be inferred from the intersections of *two*, *e.g*. orthogonal, scanning *pencil* beams (if both indicate a signal). Furthermore, (projection) absorption images along these two directions can be obtained without further exposure.

### Validation of background photon distribution

In order to experimentally validate the above solution, which is particularly based on the anisotropic distribution of the scattered photons, we have measured the flux of these background photons emitted from a 30-cm-diameter cylindrical phantom. In addition, for studying the effect of *finite* bandwidths, we utilised an industrial X-ray tube source with beam collimators and filters, delivering a pencil beam with 3 mm diameter, average photon energy of 90 keV, and 24%

**Figure 5:**
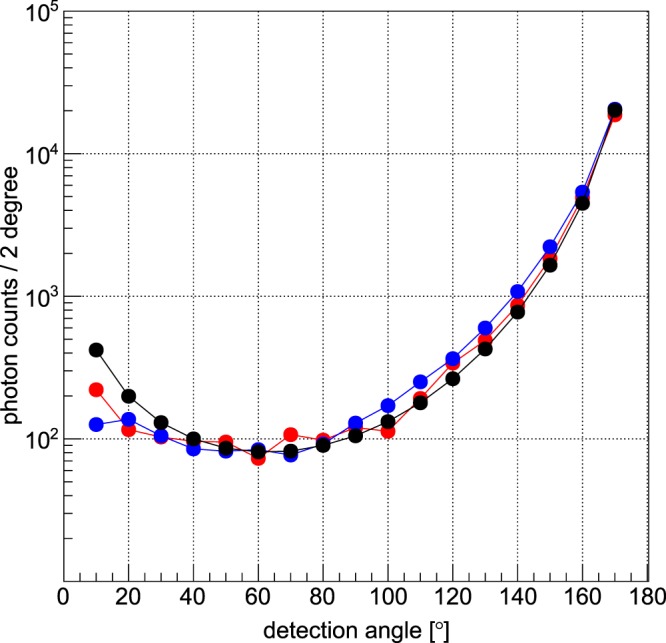
Comparison between measured, simulated, and analytically derived Compton peak heights for an unpolarised X-ray source and the 30cm-diameter phantom. Varying the detection angle Θ (same Θ as in Fig. 4) shows good agreement of the Compton-peak heights of the experiment (red), simulation (blue), and mathematical model (black). Note the logarithmic scale over more than two orders of magnitude.

**Figure 6:**
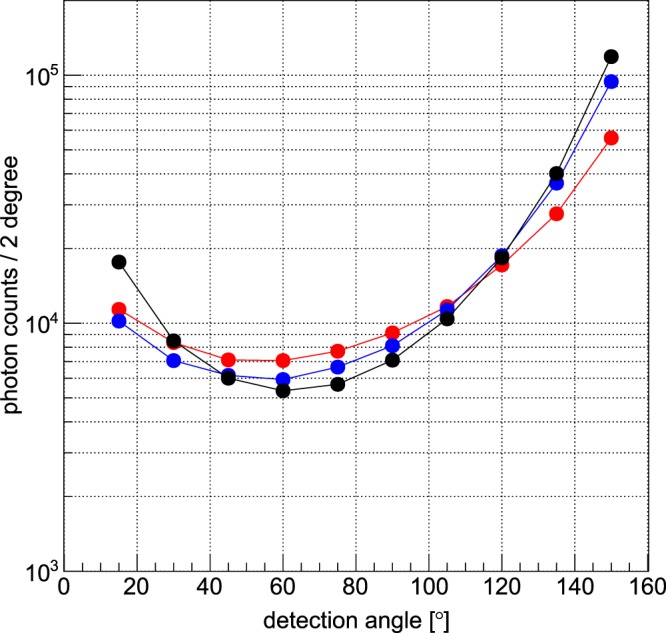
Comparison between measured, simulated, and analytically derived background photon counts in the Gold-signal region for a polarised synchrotron source and the 30cm-diameter phantom. Like in Fig. 5, also here is a good agreement of the photon counts in the signal region when experimental data (red) are compared with simulation (blue), and mathematical model (black).

Full-Width-at-Half-Maximum (FWHM) bandwidth. Figure 5 shows measured and simulated photon counts for various detection angles, as well as the comparison with estimates from a simplified mathematical model described below, exhibiting clear overall agreement. In order to validate our claim also with a monochromatic and polarised source, we have performed the same angular scan at a DESY-synchrotron-beamline with 82.8 keV photons. Figure 6 shows the comparison between the measured counts of photons in the signal region with the simulated ones, showing again a very good agreement.

This simplified model is able to explain the angular distribution of the anisotropic Compton-background and is based upon the assumption that most of the photons that are detected by any pixel have taken the shortest path from the entry point to a scatter position *z* along the incident beam direction and from there directly to the detector pixel. The model thus adds up the yields from all scattering positions along the beam direction. For each scatter position *z* we have used the (weak) angular dependence *a(z)* of Compton scattering, but neglect any energy-variation due to scattering. Furthermore, for each position *z* we take the fraction *f(z)* of the pixel’s solid angle and the projection *p(z)* of its area with respect to that position into account, as well as the total transmission *T(z)* over such shortest paths. For the polychromatic source the mean photon energy was used. We then simply sum up all contributions *y(z)* over all *z-*positions to the total yield *Y* of a given pixel and plot the result versus detector angle. Note that all mentioned functions vary only with the scatter position *z*: Y = $\sum _{{z}}y(z)$, with *y(z)* = *const ∙ a(z)∙f(z)∙p(z)∙T(z)*, where the constant takes into account all dependencies of the yield which do not depend on *z*, like the total scattering cross section, the number of incident photons, and the detector efficiency. If the distance from the scatter position *z* to the detector is called *r*, then *f(z)* = $\frac{A}{4\pi {r}^{2}}$, with the pixel area *A*, and *T(z)* = ${e}^{-\mu (z-{z}_{0}+r)}$, with µ being the (incident energy dependent) linear attenuation coefficient and *z*_0_ the entry position along the beam direction. Note that one could use also the energy dependence for the attenuation coefficient, but its variation is relatively small, which is also the case for the angular dependence and pixel area projection. As it turns out, only *T(z)* and *f(z)* vary strongly with *z*. Since the agreement with both experiments (polychromatic unpolarised X-ray tube source vs monochromatic polarised synchrotron radiation) and corresponding simulations is convincing (s. Figs 5 and 6), it seems as if the underlying principle of this simplified model reflects indeed the basic reason for the found angular distribution of the anisotropic Compton-background, namely the interplay between total path attenuation and relative solid angle which both lead to strong background yields for *large* detection angles, but “suppress” the yield for forward-angles.

**Figure 7:**
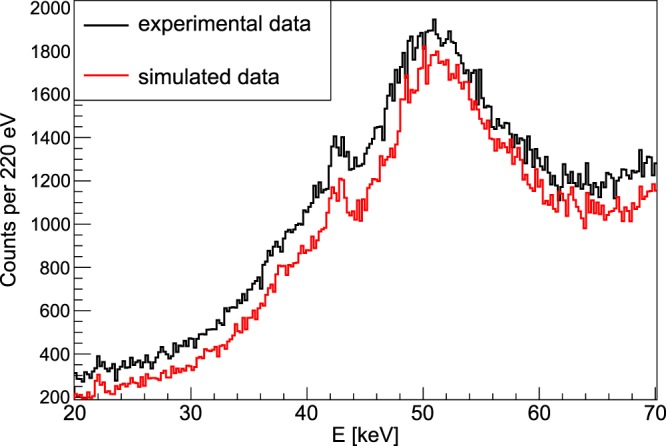
Measured and simulated spectra for XFI with a Gd-solution embedded in the 30cm-diameter phantom. The Gd-solution with a concentration of 78 mg/ml was placed within a 3 mm Eppendorf tube, embedded in the 30cm-diameter phantom. The Gd-K_α_-signal around 43 keV is clearly visible. Note that both curves are *not* scaled to each other, the difference is just 14%, attributed to the uncertainty in the photon flux measurement.

In order to also demonstrate a concrete XFI-measurement with the large 30cm-diamater phantom, we have drilled a vertical hole into the centre of it for placing a high-Z material solution in it. Since we only had a *single* detector (so to speak only *one* pixel) available with a correspondingly small effective solid angle, the concentration of the solution must be relatively large to keep measurement times low. Therefore, instead of gold solutions, we have used a Gadolinium-solution with a concentration of 78 mg/ml, which was placed within a 3 mm Eppendorf tube. Fig. 7 shows a direct comparison of the measured and simulated spectrum around the Gd-signal region. The agreement is quite convincing, when keeping in mind that both data sets are *not* scaled to each other. The (constant) difference between both by about 14% can be mainly attributed to the uncertainty in the measured incoming photon flux (determined by using a photo diode).

Although fluorescence is a non-resonant excitation process, our spatial filtering scheme shows an energy dependence, because the optimum “map” (s. *e.g*. Fig. 4b) varies slightly with energy, hence one can expect a limit for the bandwidth of the incident X-ray beam. Further simulations revealed a *critical* X-ray bandwidth of about 15% FWHM, above which the significance *Z* starts decreasing (by more than 10%). In addition, we have shown that when using the filtered spectrum of the conventional X-ray source, which was used for the data in Fig. 5, the significance *Z* drops only to 50% of the value for the (simulated) monochromatic case. Hence, our method does not require strictly monochromatic X-ray sources, but depends on sources that deliver synchrotron-like *pencil* beams.

### Translation into clinical application

While pilot studies at large-scale synchrotron facilities are helpful in developing XFI-technology towards scanning of *human* patients, their sheer size and costs preclude *clinical* applications. One pathway for translating our XFI-method into *clinics* is to use compact, laser-based, brilliant X-ray sources^16–19^. Here, electrons with relativistic energies oscillate in the field of a counter-propagating laser-pulse. Due to the relativistic energies, the X-ray emission is confined to *small* forward angles, hence allowing effective collimation for scanning *pencil* beams^32^. This collimation, together with the periodic oscillation of the electrons in the laser field, keeps the resulting bandwidths in the above-mentioned limit.

The time needed per scanning direction is fully determined by two factors: (i) the number of photons per second that the used X-ray source can generate within a pencil beam solid angle and (ii) the number of photons per second that the used detector can handle. Synchrotrons can easily produce more than the required 10^9^ photons/sec/mrad^2^ per scanning direction. Our used Amptek detector can handle an input count rate of about 50 kHz. For measurements where the X-ray beam flux was originally too large, we have used attenuators in the beamline. However, note that the larger the object the smaller the solid angle of each detector and the less restriction from the detector count rate. Our solution to the background problem requires a large area pixelated detector which could be simply realized by an arrangement of many independent detectors of the kind as used in our experiment.

## Discussion

In the present work, unprecedented gains in target tissue penetration and detection sensitivity, as well as the ability to precisely and rapidly interrogate disease-bearing tissues utilizing low-dose, molecularly-targeted GNPs, are distinct advantages offered by our novel XFI scheme for *human*-sized objects. Other XFI-schemes, which are either preferring measurements in certain *small* angle ranges and/or applying collimators with restricted fields of view on only *parts* of the entire beam volume, cannot present a low-dose solution to the intrinsic “background problem”, while our spatial filtering opens the path for the background suppression factor demanded for bringing small-animal results from tumour-diagnostics onto size-scales of future human applications. Our optimisation approach is well-justified by experiments as well as by simulations and an analytical model.

Furthermore, for a future utilisation as next-generation tool for monitoring *in vivo* pharmacokinetics, whereby the distribution of medical drugs to which GNPs are bound can be *serially* imaged, we have also studied the scaling of XFI with spatial resolution. Here, in contrast to the above-mentioned early small-tumour detection, the spatial resolution can be lowered, *e.g*. to a 1-cm-diameter sphere. In this case, the scaling of XFI-sensitivity, for the same physical dose and statistical signal significance, predicts that the minimum detectable GNP-mass inside a 1-cm-target-sphere is just 12 µg, while above it is 1.2 µg inside the 1-mm-target-sphere (both embedded in a 30-cm-diameter object), thus *lowering* the minimum detectable GNP-*concentration* by a factor of 100.

### Outlook

More than 50% of all cancer patients receive radiotherapy (RT) during their treatment regimen^33^. Despite advances in external beam RT that optimise dose delivery by *e.g*. beam shaping and real-time imaging, image-guided attenuation of therapy^34^ for treating early stage cancers and tumour recurrence^35^, a significant number of patients experience relapse, metastases, and/or significant toxicities to normal tissue^36^. Such unfavourable outcomes underscore a critical need for new probe-device combinations that can overcome these limitations, *e.g*. by enhancing RT dose to tumour while sparing normal tissues^37,38^. The use of *targeted* high-atomic number radiosensitising materials, such as the GNPs, has served as a promising solution for further increasing RT efficacy^39^. Particles, initially adapted as cancer-specific imaging probes, localise to and accumulate within tissue targets of interest - and they can be further exploited as imaging agents for image-guided X-ray RT of the target site as they boost local dose deposition to target tumour volumes^40^. Hence, GNP-based XFI would allow for combining diagnostics and therapy with one and the same device.

As a further step on the path of GNP-technology research, newer generation (*i.e*., sub 10-nm diameter) GNPs^41^ will be functionalised with tumour-targeting moieties and contrast labels to create molecularly-targeted cancer imaging probes, as in prior human clinical trials using fluorescent core-shell silica nanoparticles, referred to as Cornell dots, or simply C dots^42^. These “target-or-clear” sub-10 nm inorganic particle probes exhibit hallmarks of ideal diagnostic probes. Recent studies have shown that GNPs, ranging from 2 to 15 nm in diameter, penetrate and localise within cancer cells, multicellular spheroids, and *in vivo* tumours in a size-dependent manner^43^. Further, careful tuning of surface ligand and PEG chain densities to maintain sub-10 nm sizes has led to favourable *in vivo* biological profiles and radiation dosimetry^44^, paving the way for product translation in both surgical^45^ and other oncological settings.

XFI can also be applied to other high-atomic number atoms, *e.g*. another future application of our XFI-method could be *in vivo* measuring of Gadolinium(Gd)-retention in human brains and bones from using Gd as a contrast agent in MRI-applications. First estimates, assuming a homogeneous distribution, reveal a sensitivity of about 1 µg per gram (brain/bone) tissue. The main difference between XFI of Gd versus GNP is the lower atomic number and K-shell excitation energy. A similar application could be pharmacokinetic studies for Platinum-based chemotherapeutics (*e.g*. Cisplatin) and also here we reach the same sensitivity as in the Gd-retention case.

Summing up, for early diagnostic evaluations, including the possibility of higher resolution pharmacokinetic assessments, our spatial filtering scheme presents a low-dose solution to the “background problem” with high spatial resolution for large objects. This solution is validated by experiments and explained by a simplified analytical model. This could trigger further research activities in the fields of biomedical research and molecular imaging towards precision and personalized medicine.

## Methods

### Experimental Design

#### L1 peptides

A peptide derived from the third fibronectin type III domain of murine L1 with a lysine linker and a cysteine for coupling at the *N*-terminus was purchased from Schafer-N (Copenhagen, Denmark) and had the following sequence: H-CKKKKKPELEDITIFNSSTVLVRWRPVD-OH (L1-*N*-Lys). Poly(ethylene glycol) methyl ether with an average *M*_w_ ~ 2000 g mol^−1^ was purchased from Fluka. All other chemicals and solvents (analytical grade) were ordered from Sigma-Aldrich (Taufkirchen, Germany). Ultrapure water (Millipore, Darmstadt, Germany) was used for all procedures.

#### GNPs

The ligand *a*-methoxypoly(ethylene glycol)-*w*-(11-mercaptoundecanoate) (PEGMUA, *M* ~ 2000 g/mol) was synthesized. GNPs were synthesized by a seeded growth method; seed particles were synthesized by an optimized Turkevich protocol. GNP40s had a diameter of 37.7( ± 3.5) nm and a concentration of 0.3 nM. GNP30s had a diameter of 31.2( ± 2.0) nm and a concentration of 0.5 nM. GNP40s had a diameter of 37.7( ± 3.5) nm and a concentration of 0.3 nM. The as-synthesized GNPs were stabilized by citrate, which can be easily exchanged by thiols.

#### Functionalisation of GNPs

Peptide solutions were freshly prepared by solubilizing the peptide in water or phosphate buffered saline solution pH 7.4 (PBS). The solutions were centrifuged (13,000 g, 10 min) and the supernatants filtrated with 0.22 µm PTFE syringe filters (Carl Roth, Germany). The peptide concentrations were determined by UV/vis spectroscopy using the absorbances at 280, 320 and 350 nm. PEGMUA solutions were 1 mM in water. PEGMUA und L1-*N*-Lys were mixed in a molar ratio 3:1 and GNPs were added under stirring (500 rpm). After thorough shaking of the container, the mixtures were stirred (400 rpm) for 16 h at room temperature. In the case of GNP40s, 50,000 ligands were added per GNP, in the case of GNP30s, 100,000 ligands were added per GNP. After functionalisation the GNPs were concentrated and purified by repeated centrifugation (1,000–5,000 g, 10–15 min) and replacement of the supernatants with water. The final samples had 12 nM (~3.8 g/l) in PBS in the case of GNP40s, while GNP30s had 13 nM (~2.4 g/l) in artificial cerebrospinal fluid. A detailed description of the synthesis and characterisation of these functionalised GNPs can be found in ref.^20^.

#### Mice

Female C57BL/6 J mice were obtained from the breeding facility of the University Hospital Hamburg-Eppendorf. Mice were kept at standard laboratory conditions with food and water supply ad libitum and with an artificial 12 h light/dark cycle. All experiments were conducted in accordance with the German and European Community laws on protection of experimental animals, and all procedures used were approved by the responsible authorities of the State of Hamburg (*Behörde für Wissenschaft und Gesundheit, Amt für Gesundheit und Verbraucherschutz, Lebensmittelsicherheit und Veterinärmedizin*; animal permit numbers ORG 679 Morph and 98/09).

#### Spinal cord compression injury and tissue preparation for XFI

Spinal cord compression injury was performed as described in ref.^25^ using three-month-old female C57BL/6 J mice. Following intraperitoneal anesthesia with ketamin and xylazin (100 mg Ketanest, Parke-Davis/Pfizer, Karlsruhe, Germany, and 5 mg Rompun, Bayer, Leverkusen, Germany, per kg body weight), laminectomy at the T7–T9 spinal cord level was performed using a mouse spinal cord compression device to elicit compression force. The compression duration was controlled by an electromagnetic device: the spinal cord was maximally compressed for 1 second by a time-controlled current flow through the electromagnetic device.

Two different concentrations of L1-functionalised and non-functionalised nanoparticles in a volume of 1–2 µl were then applied via a very thin pulled-out single barrel microfilament (outside diameter <500 µm, inside diameter <300 µm, AM Systems) into the spinal cord, 0.5 mm proximal to the lesion site. This method leads to a minimal lesion injury due to the fine geometry of the microcapillary. Muscles and skin were then closed using 6–10 nylon stitches (Ethicon, Norderstedt, Germany). After surgery, mice were kept on a heated pad (37 °C) for 24–48 hours to prevent hypothermia. Injured mice were then intraperitoneally anesthetized with sodium pentobarbital (Narcoren, Merial, Hallbergmoos, Germany, 5 µl/g body weight) and transcardially perfused with 4% formaldehyde in PBS buffer. The spinal cords were isolated and post-fixed overnight at 4 °C. The tissue was then immersed in a lubricating balsam (Bayer) and subjected to XFI.

### Synchrotron-based XFI-experiments

XFI experiments of the spinal cords were performed at the Bio-imaging and Diffraction beamline P11 at DESY’s highly brilliant X-ray source PETRA III. The instrument provides linearly polarized photons tunable from 5.5 to 30 keV. Using KB mirrors, the monochromatic X-ray beam can be focused from (300·300) µm^2^ down to a size of (4·9) µm^2^ at the sample position (v × h, FWHM) with full flux from the source in the focal spot (2·10^13^ ph/s at 12 keV). To reach optimal detector dead time aluminium filters were used for flux reduction up to 0.006%. For restricting the scanning time we have chosen 0.2 mm diameter aperture for our measurements. The used Vortex-EM (Hitachi) detector is mounted perpendicular to the incident photon beam and its energy resolution is about 100 eV (rms) around the fluorescence lines. For the reconstruction of gold masses in spinal cords, a silicon waver with a (20.8 ± 0.8) nm sputtered gold layer was used as a reference target. Measured spectra were normalized by this reference and corrected by the transmission factors of the spinal cord geometry, which was approximated by a cylinder having a length of 10 mm and a radius of 1 mm. Taking inbound and outbound transmission properties into account, the optimum incident photon energy was found to be 15 keV. Gaussian peaks were used to fit background and gold fluorescence peaks. For the Compton tail, an exponential function was used.

#### ICP-MS measurements of GNP concentrations in spinal cords

For sample preparation, a microwave-assisted digestion (ETHOS.lab, MLS, Leutkirch, Germany) of the spinal cord was performed using 5 ml nitric acid (65% w/v; VWR, Fontenay-sous-Bois, France), 1 ml hydrochloric acid (32% w/v; Merck KGaA, Darmstadt, Germany) and 0.5 ml hydrogen peroxide (30% w/v; VWR, Leuven, Belgium). Digested samples were diluted with ultrapure water from a Millipore system (Direct-Q® 3 UV-R, Millipore, Bedford, MA, USA) to 20 ml for measurements. Gold nanoparticle concentrations were determined by inductively coupled plasma mass spectrometry (ICP-MS) on a 7700x instrument (Agilent Technologies, Santa Clara, CA, USA). Standard solutions were prepared from a 1 g/l gold stock solution (Merck Millipore, Billerica, MA, USA). The method detection limit was determined with 1 ng/l. It should be noted that while ICP-MS probes the total GNP-mass per spinal cord, it does not provide any spatial information. The error bars in Fig. 2 are the statistical variation from three consecutive measurements of the same probe.

#### Simulation tools

For the extrapolation onto human-sized phantoms we used simulations with the simulation toolkit GEANT4^46^. A “soft tissue” sphere with a diameter of 30 cm consisting of a GEANT4 material called “G4_TISSUE_SOFT_ICRP” (Geant4 User’s Guide for Application Developers Geant4 Collaboration Version: geant4 10.0, 6 December 2013) was considered.

The assumed detector resolution of σ_det_ = 200 eV (rms) was chosen close to the value of a real detector, that is, from the Amptek XR-100T-CdTe detector, whose resolution at 60 keV is 255 eV (rms). Note that the significance *Z* scales inversely with the square root of the detector resolution (*i.e*. a resolution of 255 eV instead of 200 eV lowers *Z* by 13%).

The radial collimator leaves started at a radius R = 30 cm, ended at R = 60 cm, and extended axially along the entire detector. The detector had a radius of 60 cm and a length of 1.60 m. The need for a collimator was met before we found out the optimum incident photon energy to be close to the K-edge. Thus, the vast majority of background photons in the signal region are Compton1(!)-photons, for which the collimator has no effect. Using a collimator is still useful to reduce the detector input count rate from multiply-Compton-scatted photons.

#### Validation experiments

We used an YXLON X-ray tube, model MG160, operated with 100 kV voltage, 20 mA current, and “N150”-filters (consisting of 4 mm aluminium and 2.5 mm tin). The pencil beam geometry was realised by a collimator with diameter of 3 mm and 30 mm length. The phantom was a cylinder made out of PMMA, with diameter of 30 cm and a height of 6 cm. We used the XR-100T-CdTe detector from Amptek, which was positioned at various angle positions directly on the phantom’s surface. Note that the simulation used the same number of incident photons as in the experiment and that we incorporated the detector geometry and the corresponding acceptance solid angle into the simulation. The synchrotron validation experiments were performed at the DESY P07 beamline, which satisfies high energy x-ray diffraction (XRD) and imaging techniques. It is tunable in the range 30 to 200 keV and optimised for micrometre focusing with Compound Refractive Lenses (CRLs). For the experiments we have chosen 4·10^8^ ph/s at 82.8 keV within a pencil beam of (100·100) µm^2^ and horizontal polarisation. The Gadolinium solution had a concentration of 78 mg/ml and was placed within a 3 mm Eppendorf tube, embedded in the 30cm-diameter phantom. Also here we have used the Amptek-detector.

In order to correct the CdTe-detector signals for the so-called hole-tailing effect, we have calibrated the detector with a series of different radioactive source covering a wide range of incident photon energies, as well as two dedicated experiments at the DESY-synchrotron, one using a direct (strongly attenuated) beam on the detector, whereby the energies are tuned in a wide range, and another one with fixed beamline energy, but different thin scatter foils of different (high-Z) materials, hence tuning the incident energy by making use of the different energies of the singly-Compton-scattered photons. With these data we have studied the parameters for mathematical functions that “smear” out the simulated raw data (with an ideal detector) into data for the realistic detector. Since today there is no large area CdTe-detector available, it is clear that so far we can only move our single detector through the full 4π solid angle, thus mimicking the situation once such a large area detector version is available. The simplest technical solution would be to place many single detectors next to each other.

### Statistical Analysis

As the signals in the experiments and simulations consisted of *two* fluorescence peaks, we decided to use *Significance* (*Z*) as a measurement of signal quality instead of the usual Signal-to-Noise ratio. This significance *Z* is calculated from the *p-value* (probability that the measured level above background is not just a fluctuation), using the number of signal photons of *both* fluorescence peaks and the number of background photons in a region of ± 3σ_det_ around the peaks, all numbers derived from Gaussian fits. Typically, *Z* is given in units of a standard deviation σ (sigma) of a normal distribution. A significance level of 3σ (*i.e*. 99.73%) was chosen to be the minimum signal strength.

## Data Availability

All raw and processed data generated in this work, including the data for the figures provided in the manuscript, are available from the corresponding author on reasonable request.
